# CXCL12-CXCR4 axis promotes the natural selection of breast cancer cell metastasis

**DOI:** 10.1007/s13277-014-1816-1

**Published:** 2014-05-09

**Authors:** Yanan Sun, Xiaoyun Mao, Chuifeng Fan, Chong Liu, Ayao Guo, Shu Guan, Quanxiu Jin, Bo Li, Fan Yao, Feng Jin

**Affiliations:** 1 Department of Breast Surgery, Department of Surgical Oncology, Research Unit of General Surgery, The First Affiliated Hospital of China Medical University, 155 Nanjing North Street, Heping District, Shenyang, Liaoning 110001 People’s Republic of China; 2 Department of Pathology, The First Affiliated Hospital and College of Basic Medical Sciences of China Medical University, Shenyang, Liaoning 110001 People’s Republic of China

**Keywords:** CXCR4, CXCL12, Breast cancer

## Abstract

CXCR4 and its ligand CXCL12 can promote the proliferation, survival, and invasion of cancer cells. They have been shown to play an important role in regulating metastasis of breast cancer to specific organs. High CXCR4 expression was also correlated to poor clinical outcome. Previous study also showed that tumor cells express a high level of CXCR4 and that tumor metastasis target tissues (lung, liver, and bone) express high levels of the ligand CXCL12, allowing tumor cells to directionally migrate to target organs via a CXCL12-CXCR4 chemotactic gradient. However, the exact mechanisms of how CXCR4 and CXCL12 enhance metastasis and/or tumor growth and their full implications on breast cancer progression are unknown. Yet it is likely that chemokine receptor signaling may provide more than just a migrational advantage by also helping the metastasized cells establish and survive in secondary environments. In this study, we investigated CXCR4 and CXCL12 expression in breast cancer and analyzed its association with clinicopathological factors by immunohistochemistry first. Then, we detected the mRNA and protein expression of CXCR4 and CXCL12 in breast cancer cell lines by Western blot and RT-PCR. The MDA-MB-231 has CXCR4 expression and very weak CXCL12 expression. So, we constructed the functional CXCL12 expression in MDA-MB-231 using a gene transfection technique. Further experiments were conducted to evaluate the effect of CXCL12 transfection on the biological behaviors of MDA-MB-231. The cell proliferation of MDA-MB-231–CXCL12 was accessed by MTT assay; the apoptosis was analyzed by an AnnexinV-FITC/propidium iodide double staining of flow cytometry method; and the cell invasive ability was examined by Matrigel invasion assay. Immunohistochemical analysis showed the co-expression of CXCR4 and CXCL12 correlated with lymph node metastasis and TNM stage (*p* < 0.01). It suggested that the chemokine CXCL12 and its sole ligand CXCR4 play important role in the malignance of breast cancer. To gain a deeper insight into it, we picked CXCR4-expressing cells MDA-MB-231 to be transfected with CXCL12 stably. The decreased cellular proliferation, increased apoptosis, and invasive ability were found in MDA-MB-231 with successful CXCL12 transfection (*p* < 0.05). Our findings underlined the CXCL12-CXCR4 axis correlated tightly with breast cancer metastasis. CXCL12-CXCR4 axis can increase the invasion and apoptosis of MDA-MB-231 simultaneously. These data strongly support the hypothesis that CXCL12-CXCR4 axis promotes the natural selection of breast cancer cell metastasis. Our findings could have significant implications in terms of breast cancer aggressiveness and the effectiveness of targeting the receptors and downstream signaling pathways for the treatment of breast cancer.

## Introduction

Breast cancer is one of the most common malignancies and the first leading cause of death and disability among women, especially young women, in low- and middle-income countries [1, 2]. Metastatic breast cancer remains the chief cause of cancer-related death among women. In tumor growth and metastasis, chemokines and their receptors have a multifaceted effect for regulating angiogenesis, tumor cell proliferation, and apoptosis, mediating tumor cell metastasis in an organ-specific manner [3]. Several groups found that CXCR4 and its ligand CXCL12 can promote the proliferation, survival, and invasion of cancer cells [4–6]. There are evidences that tumor cells express a high level of CXCR4 and that tumor metastasis target tissues (lung, liver, and bone) express high levels of the ligand CXCL12, allowing tumor cells to directionally migrate to target organs via a CXCL12-CXCR4 chemotactic gradient [7–9]. Previous study also showed the disruption of the CXCL12-CXCR4 signaling axis specifically in transformed tissue via epigenetic silencing of the chemokine ligand in colorectal carcinoma cells [10–13]. How about the role of CXCL12-CXCR4 signaling axis in breast cancer metastasis? In the present study, we investigated CXCL12 and CXCR4 expression in breast cancer and further to analyze its role in survival and invasive ability of breast cancer cells.

## Materials and methods

### Patients and tissues

Breast cancer tissue samples (*n* = 182) were obtained from surgical resection in the Department of Breast Surgical, the First Affiliated Hospital of China Medical University (China) between January 2007 and February 2013. Patients’ ages at the time of surgery ranged from 26 to 65, with an average age of 45.9 years old. Pathology classification was based on the WHO criteria published by Tavassoli et al. [14–16]. None of the patients received neoadjuvant chemotherapy or radiotherapy before operation. Clinicopathological information of each patient was reviewed using the hospital medical records.

### Ethics statement

The study protocol was reviewed and approved by the Ethics Committee of China Medical University (Shenyang, China) and of the participating hospital (the First Affiliated Hospital of China Medical University, Shenyang, China). All patients enrolled in this study have signed an informed consent form to agree to participate in this study and for publication of the results.

### Immunohistochemical staining

Formalin-fixed and paraffin-embedded specimens were cut into 4-μm-thick sections, which were subsequently dewaxed and hydrated. Immunohistochemical staining for CXCL12 (sc-28876, 1:100, Santa Cruz, USA) and CXCR4 (sc-53534, 1:500, Santa Cruz, USA) was performed using UltraSensitive™ S-P kits (Maixin-Bio, China) according to the manufacturer's instructions and using the reagent supplied with the kit. For the negative control, phosphate-buffered saline (PBS) was used in place of the primary antibodies. We adopted the German semi-quantitative scoring system in considering the staining intensity and area extent, which has been widely accepted and used in previous studies [17, 18]. Every lesions was given a score according to the intensity of the staining (no staining = 0, weak staining = 1, moderate staining = 2, strong staining = 3) and the extent of stained cells (0 % = 0, 1–10 % = 1, 11–50 % = 2, 51–80 % = 3, 81–100 % = 4; negative means 0 % area staining). The final immunoreactive score was determined by multiplying the intensity scores with the extent of positivity scores of stained cells, with the minimum score of 0 and a maximum score of 12 [17, 18]. Slides were independently examined by two pathologists. However, if there was a discrepancy in individual scores, both pathologists reevaluated together by reaching a consensus agreement before combining the individual scores. To obtain statistical results, a final score equal to or less than 1 was considered as negative, while scores of 2 or more were considered as positive.

### Breast cancer cell lines and cell culture conditions

Breast cancer cell lines including MCF-7 (low metastatic potential), MDA-MB-435s and MDA-MB-231 (high metastatic potential) were chosen for this study. They are all obtained from the American Tissue Culture Collection (Manassas, USA), and were stored in the laboratory of the Pathology Department, the First Affiliated Hospital and College of Basic Medical Sciences of China Medical University (Shenyang, China). The cells were cultured in RPMI 1640 medium (Gibco, USA) supplemented with 10 % fetal bovine serum (FBS) (Hyclone, USA) in a 5 % CO_2_ humidified atmosphere at 37 °C.

### Western blot analyses and reverse transcription-PCR (RT-PCR) analyses

The breast cells of MCF-7, MDA-MB-435s, and MDA-MB-231 were washed with ice-cold phosphate-buffered saline and then lysed in lysis buffer containing 10 mM Tris (pH 7.5), 150 mM NaCl, 10 mM ethylenediaminetetraacetic acid (EDTA), 1 % sodium dodecyl sulfate (SDS), 1 mM sodium orthovanadate, and a mixture of protease inhibitors (1 mM phenylmethylsulfonyl fluoride, 1 μg/mL pepstatin A, 2 μg/mL aprotinin). The lysates were sonicated for 10 s, centrifuged for 20 min at 20,000×*g* and then stored at −70 °C. Equal amounts (25 μg) of the cell lysates were resolved by 12 % SDS-PAGE and transferred to polyvinylidene fluoride membranes. After blocking, blots were incubated with mouse anti-CXCR4 monoclonal antibody (sc-53534, Santa Cruz, 1:500), rabbit anti-CXCL12 polyclonal antibody CXCL12 (sc-28876, 1:200, Santa Cruz, USA), or β-actin (Zhongshan Golden Bridge Biotechnology, 1:1000) overnight at 4 °C and followed by each corresponding second antibody at room temperature for 1 h at 37 °C. Then, the results developed by ECL (Pierce Biotechnology, USA). The protein bands were then analyzed using the BioImaging System (UVP, USA). The grayscale values of the CXCL12 and CXCR4 were normalized to the values of the corresponding β-actin band to determine the expression level of the protein. The experiments were repeated at least three times independently. Total RNA from MCF-7, MDA-MB-435s, and MDA-MB-231 was extracted with TRIzol reagent (Invitrogen, USA), and the quality of RNA was analyzed by A260/A280 ratio and gel analysis. The reverse transcription was performed with RNA PCR kit (AMV ver.3.0, Takara, Japan) according to the manufacturer's protocols. The sequences of primers used are as follows: CXCL12, forward, 5′-GTCAGCCTGAGCTACAGATGC-3′ and reverse, 5′-CTTTAGCTTCGGGTCAATGC-3 and CXCR4, forward, 5′-CCGTGGCAAACTGGTACTTT-3′ and reverse, 5′-GACGCCAACATAGACCACCT-3′. PCR products were electrophoresed on a 2 % agarose gel, and semi-quantitative evaluation of their mRNA expression levels was performed relative to expression of the house keeping genes glyceraldehyde-3-phosphate dehydrogenase (GAPDH). The primers for GAPDH were forward, 5′-CCACCCATGGCAAATTCCCATGGCA-3′ and reverse, 5′-TCTAGACGGCAGGTCAGGTCCACC-3′. The experiments were repeated at least three times independently. The results showed that mRNA and protein of CXCR4 were observed in MDA-MB-435s and MDA-MB-231, and mRNA and protein of CXCL12 were obvious in MDA-MB-435s and very weak in MDA-MB-231. MCF-7 has very weak CXCR4 and CXCL12 mRNA and protein expression. So, we picked MDA-MB-231 to be transfected with CXCL12, and further to investigate the role of CXCL12 in the MDA-MB-231 with CXCR4 expression.

### CXCL12 stable transfection

The human full-length CXCL12 cDNA fragment was ligated to the cloning site of pIRES2-ZsGreen1 (Invitrogen, USA), followed by transformation using One Shot E.coli (Invitrogen, USA) verification and amplification. Purified plasmid, or control plasmid, was used to transfect MDA-MB-231 cells by electroporation using an electroporator and EasyJet Plus, followed by selection with G418 (Sigma, Germen). Stable CXCL12 transfectant (MDA-MB-231–CXCL12), or stable control plasmid transfectant (MDA-MB-231–ZsGreen1), was subsequently established and verified (RT-PCR and Western blot).

### MTT assay

Cell proliferation of MDA-MB-231–CXCL12 and MDA-MB-231–ZsGreen1 was assessed at various time points by 3-(4,5-dimethylthiazol-2-yl)-2,5-diphenyltetrazoliumbromide (MTT) assay. The wild-type MDA-MB-231 served as the control. Briefly, 2,000 cells were seeded in each well of a 96-well plate (eight repeats) and allowed to adhere for 8 h. Then, 5 mg/ml MTT (Sigma, Germen) was added to each well and incubated for 4 h. The cells were lysed by adding 150 μl/well of dimethyl sulfoxide and read at 490 nm absorbance wavelength in microplate reader. The experiments were repeated at least three times independently.

### Flow cytometry apoptosis assay

Analysis of apoptosis was accessed by an AnnexinV-FITC/propidium iodide double staining kit (Genmed Bioscience, China) following the manufacturer’s protocols. Briefly, the wild-type MDA-MB-231, MDA-MB-231–CXCL12, and MDA-MB-231–ZsGreen1 were plated in six wells. Of BVDU, 1 μM was treated. Cells were continuously cultured for 48 and 72 h, and then to be harvested. Before reading on the flow cytometer, cell suspensions were washed in PBS, resuspended with a 1 × binding buffer and exposed to 5 μL of Annexin V-FITC (20 μg/mL) and 10 μL of propidium iodide (PI; 50 μg/mL). After incubation of 20 min in the dark, the samples were subjected to a FACScan flow cytometer [equipped with CellQuest and ModFITLT for Mac V1.01 software (Becton Dickinson, San Jose, CA)]. The experiments were repeated at least three times independently.

### Matrigel invasion assay

Cell invasive ability was examined using a 24-well Transwell with 8.0-μm pore polycarbonate membrane inserts (Corning Inc., NY, USA) according to the manufacturer’s protocol. The Matrigel (100 μl/ml) was applied to the upper surface of the membranes. After the treatment of transfection for 48 and 72 h, cells of MDA-MB-231–CXCL12 and MDA-MB-231–ZsGreen were seeded on the upper chamber (5 × 10^4^ cells/well) and incubated for 18 h. Cells that had invaded the surface of the membrane were fixed with methanol and stained with hematoxylin. The cells that invaded and moved onto the lower surface of the filter membrane were counted in 10 random high power fields (×400) by an inverted microscope. The experiment was repeated five times, and the data were shown in mean ± standard deviation (SD).

### Statistical analysis

SPSS version 13.0 for Windows was used for all analyses. The Pearson chi-Square test was used to analyze the relationship between CXCL12 and CXCR4 expression with clinicopathological factors in breast cancer. One-way analysis of variance (ANOVA) was performed to compare data from the densitometry analysis of MTT, FAS, Western blotting, and RT-PCR and Matrigel invasion assay. Statistical significance in this study was set at *p* < 0.05. All reported *p* values are two-sided.

## Result

### Expression of CXCL12 and CXCR4 in breast cancer

**Fig. 1:**
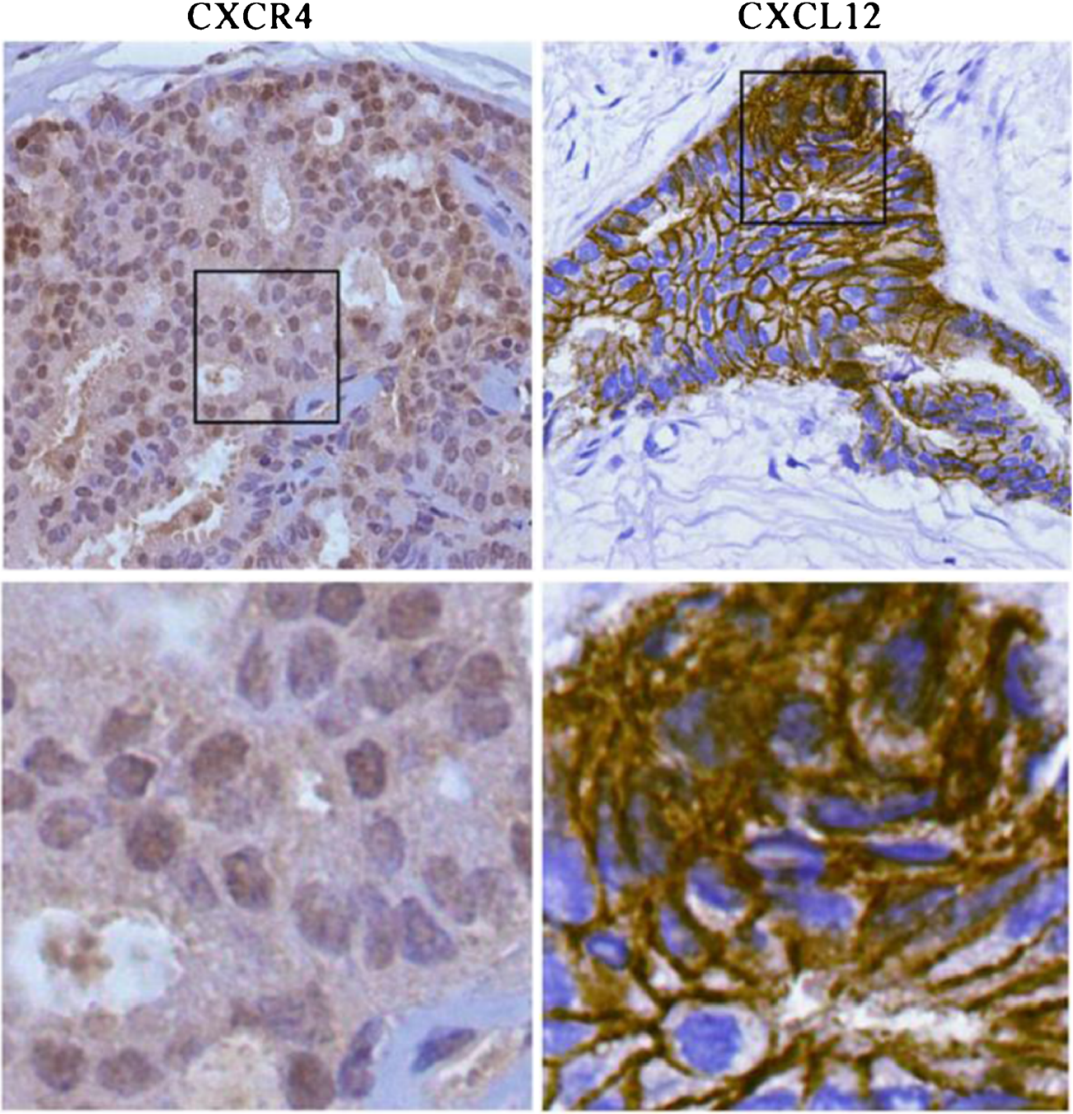
Representative immunohistochemical staining for CXCL12 and CXCR4 in breast cancer. Positive CXCL12 immunostaining was seen in invasive breast cancer; the patterns of CXCL12 expression were mixed nuclear/cytoplasmic staining. Positive CXCR4 immunostaining was seen in invasive breast cancer; the patterns of CXCR4 expression were mixed membrane/cytoplasmic staining. Original magnification, all ×200

The results of IHC revealed the membrane and cytoplasmic staining of the CXCR4 protein (Fig. 1) and the nuclear and cytoplasm staining of the CXCL12 protein in breast cancer (Fig. 1). The positive CXCR4 protein expression was seen in 83/182 (45.6 %) cases of breast cancer and CXCL12 positive expression in 71/182 (39.0 %) breast cancer cases. The relationship between CXCL12 and CXCR4 expression and different clinicopathological factors in breast cancer is shown in Table 1. CXCR4 expression was significantly associated with lymph node metastasis and TNM stage (0.01 < *p* < 0.05), but not with age, menopausal status, first term birth age, tumor size, and tumor stage (*p* > 0.05). CXCL12 expression correlated with TNM stage (0.01 < *p* <0.05). The results of immunohistochemistry demonstrated CXCR4 and CXCL12 co-expression in 27.4% (50/182) breast cancer samples and the co-expression of CXCR4 and CXCL12 correlated with lymph node metastasis and TNM stage (*p* < 0.01) (Table 2). It suggested that the chemokine CXCL12 and its sole ligand CXCR4 played important role in the malignant of breast cancer.

**Table 1:** CXCL12 and CXCR4 expression in 182 patients with breast cancer

Characteristics	Number	CXCL12 expression		*p* value	CXCR4 expression		*p* value
		Positive	Negative		Positive	Negative	
		*n* = 71	*n* = 111		*n* = 83	*n* = 99	
Age							
<45 years	101	40	61	*χ*2 = 0.03	50	51	*χ*2 = 1.39
≥45 years	81	31	50	*p* > 0.05	33	48	*p* > 0.05
Menopausal status							
Premenopausal	135	50	85	*χ*2 = 0.85	60	75	*χ*2 = 0.28
Postmenopausal	47	21	26	*p* > 0.05	23	24	*p* > 0.05
Tumor size							
<2 cm	83	36	47	*χ*2 = 1.22	43	40	*χ*2 = 2.37
≥2 cm	99	35	6	*p* > 0.05	40	59	0.01 < *p* < 0.05
Lymph node metastasis							
Negative	112	53	59	*χ*2 = 0.35	45	67	*χ*2 = 3.21
Positive	70	30	40	*p* > 0.05	38	32	*p* > 0.05
Tumor stage							
I + II	140	49	91	*χ*2 = 4.10	58	82	*χ*2 = 4.26
III + IV	42	22	20	0.01 < *p* < 0.05	25	17	0.01 < *p* < 0.05

**Table 2:** Co-expression of CXCL12 and CXCR4 in 182 patients with breast cancer

Characteristics	Number	Co-expression of CXCL12 and CXCR4		*p* value
		Positive	Negative	
		*n* = 50	*n* = 132	
Age				
<45 years	101	32	69	*χ*2 = 2.02
≥45 years	81	18	63	*p* > 0.05
Menopausal status				
Premenopausal	135	35	100	*χ*2 = 0.63
Postmenopausal	47	15	32	*p* > 0.05
Tumor size				
<2 cm	83	19	64	*χ*2 = 1.95
≥2 cm	99	31	65	*p* > 0.05
Lymph node metastasis				
Negative	112	23	89	*χ*2 = 7.03
Positive	70	27	43	*p* < 0.01
Tumor stage				
I + II	140	30	110	*χ*2 = 11.12
III + IV	42	20	22	*p* < 0.01

### The CXCL12 and CXCR4 expression in MDA-MB-435s and MDA-MB-231

**Fig. 2:**
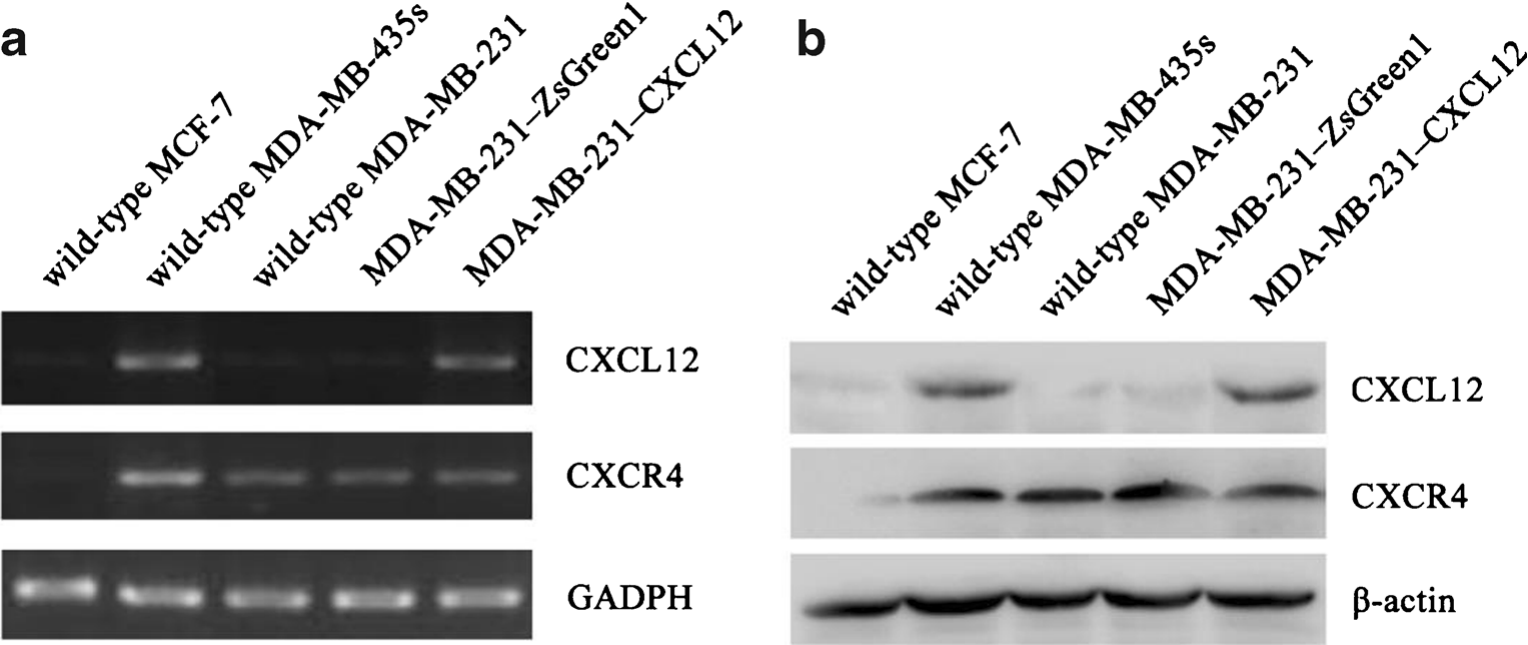
The expression patterns of CXCL12 and CXCR4 in breast cancer cell lines. Total RNA and proteins were extracted from wild-type MCF-7, wild-type MDA-MB-435s, wild-type MDA-MB-231, MDA-MB-231–CXCL12 and MDA-MB-231–ZsGreen1. Then they were subjected to RT-PCR and western blot analysis, respectively, for CXCL12 and CXCR4. Both mRNA (**a**) and protein (**b**) expression of CXCR4 and very weak expression of CXCL12 were observed consistently in MDA-MB-231. CXCR4 and CXCL12 mRNA (**a**) and protein (**b**) expression was found in MDA-MB-435s. MCF-7 has very weak CXCR4 and CXCL12 mRNA and protein expression (**a** and **b**). So we picked MDA-MB-231 to be transfected with CXCL12. CXCL12 mRNA (**a**) and protein (**b**) expression was observed in MDA-MB-231–CXCL12 cells, but very weak in MDA-MB-231–ZsGreen1 or wild-type MDA-MB-231 cells. It is indicated that the stable CXCL12 transfection in MDA-MB-231 was successfully constructed

As shown in Fig. 2, the mRNA and protein of CXCR4 were observed in MDA-MB-435s and MDA-MB-231, CXCL12 expression were observed in MDA-MB-435s, and its expression was very weak in MDA-MB-231. MCF7 has very weak CXCR4 and CXCL12 mRNA and protein expression (Fig. 2). So, we picked MDA-MB-231 to be transfected with CXCL12.

### CXCL12 transfection in MDA-MB-231

MDA-MB-231 was transfected with pIRES2-ZsGreen1 plasmid encoding human CXCL12 or empty vector pIRES2-ZsGreen1 as a control, followed by selection with G418 to yield a stable CXCL12-expressing cell line (MDA-MB-231–CXCL12) and a stable control plasmid transfectant (MDA-MB-231–ZsGreen1). Using Western blot and RT-PCR analysis, CXCL12 protein expression was observed in MDA-MB-231–CXCL12 cells, but very weak in MDA-MB-231–ZsGreen1 or wild-type MDA-MB-231 cells (Fig. 2). It is indicated that the stable CXCL12 transfection in MDA-MB-231was successfully constructed.

### Effect of CXCL12 transfection on cell proliferation by MTT

**Fig. 3:**
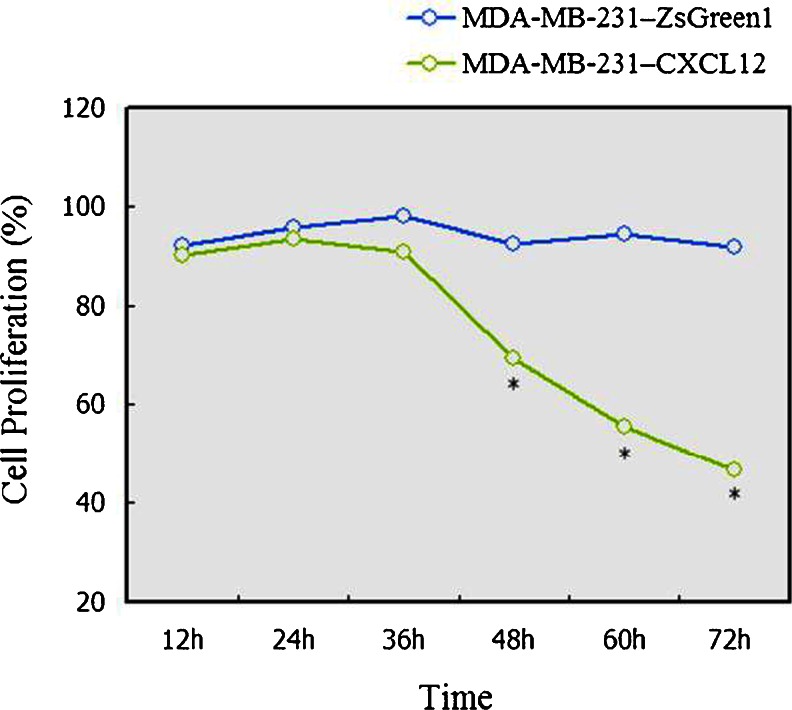
The proliferation curve of MDA-MB-231–CXCL12 and MDA-MB-231–ZsGreen1 was constructed by plotting absorbance against time using the MTT method. *Values* represent the mean ± SD of five independent experiments. The cell proliferation rate of MDA-MB-231–CXCL12 cells was markedly lower than that of MDA-MB-231–ZsGreen1 or wild-type MDA-MB-231 cells at 48 h, 60 h, and 48 h (*p* < 0.05). ^*^indicates a statistically significant difference between MDA-MB-231–CXCL12 group and MDA-MB-231–ZsGreen1 group (*p* < 0.05)

The cell proliferation rate of MDA-MB-231–CXCL12 cells was markedly lower than that of MDA-MB-231–ZsGreen1 or wild-type MDA-MB-231 cells at 48, 60, and 72 h (*p* < 0.05) as shown by the MTT results and proliferation curve (Fig. 3), indicating that the CXCL12/CXCR4 expression induced the decreased cellular proliferation.

### CXCL12 transfection up-regulated the apoptosis of MDA-MB-231

**Fig. 4:**
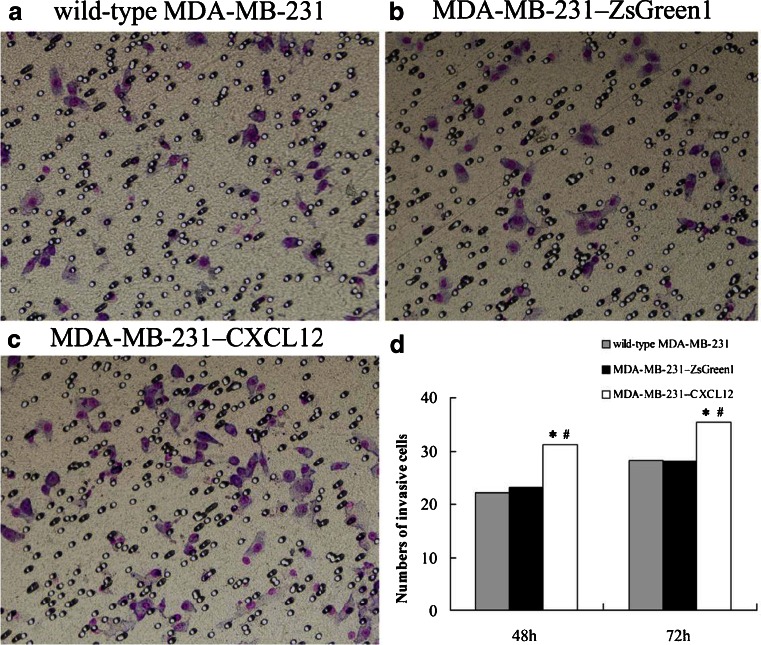
The CXCL12 transfection increased the invasive ability of breast cancer cell MDA-MB-231. Matrigel invasion assay showed that the invasive ability of the wild-type MDA-MB-231 (**a**), MDA-MB-231–ZsGreen1 (**b**) and MDA-MB-231–CXCL12 (**c**) at 48 h after transfection (**d**) Number of cells invading onto the lower surfaces of the filter was counted. Data represented the mean±SD of three independent experiments, *p<0.05 vs. wild-type MDA-MB-231 group, # *p*<0.05 vs. MDA-MB-231–ZsGreen1 group

We monitored the level of apoptosis induction by FCM. As shown in Fig. 4, MDA-MB-231 transfected with CXCL12 showed different percentage of apoptosis with wild-type MDA-MB-231 or MDA-MB-231–ZsGreen1 at 48 and 72 h after transfection. Apoptotic cells ratio of MDA-MB-231–CXCL12 was present 32.7 % at 48 h and 41.1 % at 72 h after transfection, which was higher than the MDA-MB-231–ZsGreen1 (15.0 % at 48 h after transfection, 12.3 % at 72 h after transfection) or the wild-type MDA-MB-231 group (13.5 % at 48 h after transfection, 16.8 % at 72 h after transfection) (*p* < 0.05).

### CXCL12 transfection increased invasive ability of MDA-MB-231

Matrigel invasion assays showed that the invasive ability of the MDA-MB-231 cell was enhanced with CXCL12 transfection after the treatment of transfection for 48 and 72 h. The MDA-MB-231–ZsGreen1 and wild-type MDA-MB-231 had fewer numbers of cell which invaded onto the lower surfaces of the Transwell filters than MDA-MB-231–CXCL12 (*p* < 0.05).

## Discussion

Breast cancer is a major public problem; according to the World Health Organization, more than 1.2 million people will be diagnosed each year with breast cancer worldwide; breast cancer will be responsible for 3 % of deaths among women [19, 20]. Over the past decade, reports have demonstrated that chemokines and their receptors play critical roles in the development of cancer, including tumor cell growth, migration, and immune cells in a tumor [21–24]. Tumor metastasis is also a non-random, highly organ-specific pathophysiological process. A growing body of evidence has indicated that the chemokine CXCL12 and its cognate receptor CXCR4 have a critical role in this process [25–27]. One such chemokine is the ubiquitous CXCL12 which signals via the chemokine receptors CXCR4. The CXCR4-CXCL12 signaling axis contributes to metastasis and clinical outcomes in many different types of solid cancer, including breast cancer [28–33]. This receptor ligand pair appears to be involved in the directed migration of cancer cells to sites of metastasis, increased survival of cancer cells in sub-optimal conditions, and establishment of a tumor promoting cytokine/chemokine network. In the present study, we demonstrated that the co-expression of CXCR4 and CXCL12 was correlated with lymph node metastasis and TNM stage of breast cancer, CXCR4 expression was significantly associated with lymph node metastasis and TNM stage, and CXCL12 expression was found to be correlated with TNM stage. A number of functional studies investigating the influence of CXCR4 expression and activation by its ligand CXCL12 have recently been performed, revealing that CXCR4-CXCL12 axis plays an important role in regulating metastasis of breast cancer to specific organs [23, 25, 34, 35]. A lot of previous studies indicated that CXCR4 expression played a crucial role in breast metastasis and correlated with poor clinical outcome. The previous views also suggested that tumor cells express high CXCR4, and the metastasis-targeted organs express high CXCL12, so the tumor cells were attracted to the ligand in these organs [36]. Given this dichotomy between the physiologic and pathophysiologic functions of CXCR4, we hypothesized the constitutive expression of CXCL12 may play a pivotal role in determining the function of CXCR4 signaling in the human breast cancer. Wendt et al. reported that the expression of CXCL12 induces anoikis in colorectal carcinoma cells, and silencing CXCL12 within colonic carcinoma cells greatly enhances their metastatic potential. Their research suggested that physiologic, endogenous expression of CXCL12 limits tumor metastasis and participates in the homeostatic turnover of intestinal epithelial cells [10, 12]. Whether the CXCR4-CXCL12 promotes or limits the breast cancer metastasis?

To gain a deeper insight into the role of it in breast cancer, we investigated the CXCL12 and CXCR4 expression in breast cancer cells MCF-7, MDA-MB-435s, and MDA-MB-231. The results of Western blot and RT-PCR showed that mRNA and protein of CXCR4 were observed in MDA-MB-231, and mRNA and protein of CXCL12 were obvious in MDA-MB-435s and very weak in MDA-MB-231. So, we picked MDA-MB-231 to be transfected with CXCL12. Our result showed that the MDA-MB-231–CXCL12 displayed apparent CXCL12 expression, whereas MDA-MB-231–ZsGreen1 showed absent CXCL12 expression at the RNA and protein level. We accessed that the expression of CXCL12 limited the cellular proliferation and promoted its apoptosis. Interestingly, we also accessed high invasion in MDA-MB-231 coupled with stable CXCL12 transfection. Are they contradictory? Our previous research indicated that co-expression of CXCR4 and CXCL12 was correlated with lymph node metastasis and TNM stage of breast cancer. How do we explain it? It seemed the limited cellular proliferation and promoted apoptosis are not opposite to increased invasive ability.

Previous study indicated that the CXCR4-CXCL12 signaling axis contributes to metastasis and clinical outcomes in breast cancer. Supporting these data, our clinicopathological study revealed that patients with co-expression of CXCR4 and CXCL12 tend to have positive lymph node metastasis and clinical progression of TNM stage, suggesting that CXCR4-CXCL12 axis is a significant prognostic marker in breast cancer patients. The high invasion in MDA-MB-231 coupled with stable CXCL12 transfection also supported these data. The limited cellular proliferation and promoted apoptosis doesn’t opposite to the increased invasive ability. Our experiments about MDA-MB-231 were repeated at least three times independently; we got the result of the same trend. Wendt et al. found that the constitutive expression of CXCL12 in human colorectal carcinoma cells reduced orthotopic tumor formation and inhibited metastasis in severe combined immunodeficient mice. And CXCL12 expression induced apoptosis specifically in nonadherent colorectal carcinoma cells. They considered the autocrine expression of CXCL12 also accelerated cellular detachment which coupled with breakdown of the focal adhesion complex and dependent upon alterations in laminin in the extracellular matrix. The coupling of increased cell shedding and sensitivity to cell death following detachment from the substratum thoroughly recapitulates the anoikis process of normal intestinal epithelium [10, 12]. We thought the stably CXCL12 transfection can change the behavioral biology of cancer cells. The cell proliferation does not equate cell migration. They had tight relation with each other. The limited cellular proliferation and promoted apoptosis are not opposite to increased invasive ability. The stably CXCL12 transfection coupled with CXCL12-CXCR4 axis can promote the invasion of MDA-MB-231, increasing the apoptosis of MDA-MB-231 simultaneously. More powerful and malignant cells survive via CXCL12-CXCR4 axis. Tumor metastasis is the process of natural selection of cancer cells. It was the survival of the fittest. We speculated that the CXCL12-CXCR4 axis can promote the natural selection of breast cancer cell metastasis. The co-expression of CXCR4 and CXCL12 which correlated with lymph node metastasis and TNM stage of breast cancer also has a reasonable reason.

In summary, the results of this study suggest that CXCL12-CXCR4 axis was correlated tightly with breast cancer metastasis. CXCL12-CXCR4 axis can promote the invasion of MDA-MB-231, increasing the apoptosis of MDA-MB-231 simultaneously. The CXCL12-CXCR4 axis can promote the natural selection of breast cancer cell metastasis, so the adaptive malignant cells can survive. Blocking CXCR4/CXCL12-induced signaling can slow down the breast cancer growth and metastasis.
